# Draft Genome Sequence of Pseudomonas aeruginosa Strain BWH047, a Sequence Type 235 Multidrug-Resistant Clinical Isolate Expressing High Levels of Colistin Resistance

**DOI:** 10.1128/MRA.00623-19

**Published:** 2019-07-18

**Authors:** Kelly E. R. Bachta, Egon A. Ozer, Alisha Pandit, Francisco M. Marty, John J. Mekalanos, Alan R. Hauser

**Affiliations:** a Department of Microbiology-Immunology, Northwestern University, Chicago, Illinois, USA; b Department of Medicine, Division of Infectious Diseases, Northwestern University, Chicago, Illinois, USA; c Division of Infectious Disease, Brigham and Women’s Hospital, Boston, Massachusetts, USA; d Department of Microbiology, Harvard Medical School, Boston, Massachusetts, USA; University of Arizona

## Abstract

The Gram-negative bacterium Pseudomonas aeruginosa is often multidrug resistant, associated with global epidemic outbreaks, and responsible for significant morbidity and mortality in hospitalized patients. Here, we present the draft genome sequence of BWH047, a multidrug-resistant P. aeruginosa clinical isolate belonging to the epidemic sequence type 235 and demonstrating high levels of colistin resistance.

## ANNOUNCEMENT

Pseudomonas aeruginosa frequently causes severe health care-associated infections (1) that are associated with high mortality. Multidrug-resistant (MDR) P. aeruginosa infections are consistently associated with poor clinical outcomes (2). The global P. aeruginosa population includes several recognized high-risk epidemic clones belonging to multilocus sequence type 111 (ST-111), ST-175, or ST-235 (3, 4). ST-235, a globally distributed multidrug-resistant high-risk clone associated with nosocomial infections, is known to harbor a variety of antimicrobial resistance genes, including those for β-lactamases and carbapenemases, as well as horizontally acquired antibiotic resistance elements (4).

In this study, we determined the draft genome sequence of the ST-235 MDR P. aeruginosa clinical isolate BWH047, an isolate collected from a patient at Brigham and Women’s Hospital (BWH) in 2016. BWH047 was isolated from a bronchoalveolar lavage fluid specimen from an immunosuppressed 57-year-old patient who had undergone allogeneic hematopoietic cell transplantation in 2009 and bilateral lung transplantation in April 2015. After persistent recovery of P. aeruginosa with escalating antimicrobial resistance, inhaled colistin therapy was initiated in August 2015. Antibiotic susceptibilities were determined by Kirby-Bauer (KB) testing (5). BWH047 was initially sensitive to amikacin, cefepime, ceftazidime, colistin, and piperacillin, resistant to ciprofloxacin, levofloxacin, gentamicin, tobramycin, and meropenem, and intermediate to aztreonam. Broth microdilution (MBD) resistance testing (6) was completed, and BWH047 was found to be sensitive to aztreonam, ceftazidime, and piperacillin-tazobactam, intermediate to cefepime, and resistant to ciprofloxacin, colistin, gentamicin, and meropenem. Despite initial KB testing reports of colistin sensitivity, MBD testing demonstrated considerable colistin resistance.

Genomic DNA isolated from a single colony of BWH047 was sequenced on the HiSeq 4000 platform (Illumina, Inc., San Diego, CA), generating 2 × 150-bp paired-end reads. A total of 4,957,758 reads were generated, for a total of 743,663,700 bases. Adapter sequences were removed, and reads were quality trimmed using Trimmomatic v0.36 (7), resulting in 3,941,146 paired reads totaling 569,414,246 bases. *De novo* assembly was performed using SPAdes v3.9.1 (8). Quality control was performed by aligning trimmed reads to assembly contigs using bwa v0.7.15 (9). All contigs shorter than 200 bp or with an average fold coverage of <5× per base were removed. The resulting assembly consisted of 138 contigs totaling 6,742,224 bases of sequence, and the average fold coverage was 84×. The assembly *N*_50_ value was 192,392 bp, and the average GC content was 66.15%. Annotation was performed using the NCBI Prokaryotic Genome Annotation Pipeline v4.8 (10, 11) and identified 6,368 coding sequences. Default parameters were used for all software, unless otherwise specified.

To examine the antibiotic resistance profile of BWH047, antibiotic resistance genes were identified using ResFinder v3.1.0 (12) and the CARD database v3.0.1 (13). We identified two β-lactamase genes, *bla*_OXA-488_ and PDC-2 (14). Also identified were two aminoglycoside resistance genes [*aph*(*3′*)-*IIb* and *ant*(*2″*)-*Ia*], a single nucleotide insertion in *oprD* resulting in a frameshift in the C-terminal portion of the gene likely conferring carbapenem resistance, mutations in *gyrA* (T83I) and *parC* (S87L) conferring fluoroquinolone resistance, one fosfomycin resistance gene (*fosA*), one phenicol resistance gene (*catB7*), one sulfonamide resistance gene (*sul1*), and one trimethoprim resistance gene (*dfrA10*). No canonical colistin resistance mutations were observed (15), and homology to plasmid-borne colistin resistance genes *mcr1* to *mcr9* was not detected (16, 17). All identified resistance genes had a nucleotide identity of 97.47 to 100% over 85 to 100% of the reference gene lengths.

### Data availability.

Illumina sequencing reads were deposited in the NCBI Sequence Read Archive (SRA) with accession numbers PRJNA533806 and SRR9109023. This whole-genome shotgun project has been deposited at DDBJ/ENA/GenBank under the accession number SUPJ00000000. The version described in this paper is version SUPJ01000000.
